# Dietary Acid Load Was Positively Associated with the Risk of Hip Fracture in Elderly Adults

**DOI:** 10.3390/nu14183748

**Published:** 2022-09-10

**Authors:** Cheng-Feng Li, Yu-Ping Liu, Chun-Ying Liu, Hui-Lian Zhu, Bao-Hua Wu, Bao-Lin Li, Yu-Ming Chen

**Affiliations:** 1 Department of Epidemiology, School of Public Health, Sun Yat-sen University, Guangzhou 510080, China; 2 Department of Nutrition, School of Public Health, Sun Yat-sen University, Guangzhou 510080, China; 3 Guangzhou Orthopaedics Trauma Hospital, Guangzhou 510045, China

**Keywords:** dietary intake, acid load, bone, fracture, case–control study

## Abstract

Previous studies have shown that dietary acid load (DAL) harms bone health, but the evidence is inconsistent and insufficient. This study examined the relationships between DAL and the risk of hip fracture. This case–control study contained 1070 pairs of 1:1 age-, city-, and gender-matched incident cases and controls (mean age, 71 years) recruited in Guangdong, China. Dietary information was collected using a validated 79-item food frequency questionnaire through face-to-face interviews. DAL was estimated based on established algorithms for the potential renal acid load (PRAL) and net endogenous acid production (NEAP). Higher PRAL and NEAP were dose-dependently associated with a higher risk of hip fracture in both the conditional logistic regression model and restricted cubic spline analysis after adjusting for potential covariates. The multivariate-adjusted odds ratios and 95% CI of hip fracture for tertiles 2 and 3 (vs. 1) of DAL were 1.63 (1.18, 2.25) and 1.92 (1.36, 2.71) for PRAL and 1.81 (1.30, 2.53) and 2.55 (1.76, 3.71) for NEAP in all participants (all *p*-trends < 0.001), respectively. Subgroup analyses showed more pronounced associations in participants with a lower body mass index. Our findings suggested positive associations between the estimated DAL and the risk of hip fractures in the elderly Chinese population.

## 1. Introduction

Osteoporosis has been regarded as an important global public health concern, especially in the elderly [1,2], and it causes huge disease and economic burdens worldwide [3]. Hip fracture is deemed the most devastating result of osteoporosis owing to its high disability and mortality rates [4,5]. Effective preventive strategies are, therefore, imperative for controlling hip fractures and their related adverse consequences.

A growing number of studies have suggested that dietary factors are closely related to bone mineral density (BMD) [6,7]. It was reported that adherence to the 2006 American Heart Association Diet and Lifestyle Recommendations (AHA-DLR) [8] or the Mediterranean dietary pattern was associated with better bone health [9,10]. However, a Western diet is characterized by the high consumption of animal protein and low intake of fruit and vegetables. It might lead to chronic metabolic acidosis, which is linked to a negative impact on bone health [11,12,13].

Dietary acid load (DAL), commonly assessed by the potential renal acid load (PRAL) and the net rate of endogenous non-carbonic acid production (in brief, net endogenous acid production, NEAP), is often used to evaluate the acid–base equilibrium state of the body [14]. NEAP considers the ratio of intakes of protein/potassium [11], while PRAL evaluates the balance between total intake of protein and phosphorus and the consumption of potassium, magnesium, and calcium [15]. Several studies have reported that high DAL had detrimental associations with BMD and fracture risk [16,17,18,19,20,21,22], but the results remain controversial [23]. Interestingly, the majority of the studies that reported no significant associations had cross-sectional designs, small sample sizes, or Caucasian populations [24,25,26]. Nevertheless, it is worth mentioning that studies have demonstrated that there are differences in bone structure among various races [27,28]. Moreover, limited studies were conducted among Asians with notably different dietary patterns from Westerners. Therefore, we hypothesized that Asian traditional dietary patterns characterized by more plant foods and fewer animal foods have a lower dietary acid load that would contribute to a lower risk of hip fracture in Asians. On the basis of the underlying limitations described above, accumulating epidemiological evidence is warranted in this area. Furthermore, to our knowledge, despite the popularity of recent studies on the relationship between DAL and metabolic diseases (such as cardiovascular disease and kidney disease) [29,30,31], epidemiological studies directly examining the relationship between DAL and hip fracture are still inadequate.

In the current study, we examined the association between DAL and the risk of hip fracture among middle-aged and older people using data from a relatively large-scale case–control study in Guangdong Province, China.

## 2. Materials and Methods

### 2.1. Study Population

This 1:1 matched case–control study was carried out between 2009 and 2015 in Guangdong Province, China. The detailed eligibility criteria and recruitment methods have been described previously [32]. In brief, eligible patients aged 52–83 years who had lived in Guangdong Province for more than ten years were recruited. They were patients with newly diagnosed (within the previous two weeks) hip fractures confirmed by X-ray and caused by low-energy trauma at the femoral neck or intertrochanteric region. All patients were recruited from four hospitals in Guangdong Province, south China: Guangzhou Orthopedics Trauma Hospital, Guangdong General Hospital, The First Affiliated Hospital of Sun Yat-Sen University in Guangzhou City, and The Orthopedics Hospital of Baishi District in Jiangmen City, Guangdong Province. The exclusion criteria were as follows: (i) high-energy fractures (e.g., car accidents, falling from above a height of 2 m, or other strong external forces/trauma) or pathological fractures (e.g., bone tumors, osteomyelitis, bone tuberculosis); (ii) dietary habits significantly changed in the preceding five years; (iii) a history of hospital-confirmed chronic diseases that may notably influence dietary habits or bone health, including coronary heart disease, diabetes, liver cirrhosis, cancer, cognitive disorders, gastric or intestinal excision, renal failure, thyroid disorders, chronic diarrhea, or physically disabled and unable to live a normal life; (iv) current use of certain medications such as thiazide diuretics, exogenous estrogens, or corticosteroids; and (v) poor vision. A total of 1070 eligible patients were enrolled in our study.

Each case was matched with an appropriate control based on age (±3 years), sex, and city of residence. The same inclusion and exclusion criteria were applied to the control participants, except for a history of fractures. The controls were recruited from either community (82.9%) or hospitals (17.1%). Community-based controls were apparently healthy and recruited through various strategies, such as flyers, written invitations, or referrals. Hospital-based controls were newly admitted (within the previous weeks) inpatients due to certain diseases unlikely to have had notable dietary changes, such as pneumonia, influenza, and benign tumors.

### 2.2. Dietary Assessments and Calculation of DAL

A validated semiquantitative 79-item food frequency questionnaire (FFQ) was used to evaluate habitual dietary information in the last year. A quantified food atlas and commonly used utensil sizes (bowls, cups, spoons, etc.) were used to facilitate dietary intake estimation during the face-to-face interviews by research staff with medical knowledge. The participants were asked to report their habitual amounts and frequencies of food consumption (per day, week, month, year, or never). The dietary intakes of certain nutrients, including energy, protein, fat, carbohydrates, calcium, phosphorus, vitamin D, sodium, magnesium, and potassium, were calculated according to the Chinese Food Composition Table 2009. The degree of saltiness of usual homemade foods was evaluated to five degrees. The alternate Mediterranean diet score (aMed) was calculated [33] as a dietary quality score. The dietary acid load was estimated by both PRAL [15] and NEAP [11] based on the following established algorithms:

NEAP (mEq/d) = 54.5 × (protein (g/d)/potassium (mEq/day)) − 10.2(1)

PRAL (mEq/d) = 0.49 protein (g/d) + 0.037 phosphorus (mg/d) − 0.021 potassium (mg/d) − 0.026 magnesium (mg/d) − 0.013 calcium (mg/d)(2)

### 2.3. Covariates

Body mass index (BMI) was calculated from measurements of weight and height (kg/m^2^) with light clothes and no shoes. Face-to-face interviews using pretested and structured questionnaires were conducted to collect basic information on socio-demographic (age, sex, occupation, educational attainment, marital status, family income) and lifestyle (smoking, passive smoking, alcohol and tea drinking, physical activity, and healthy lifestyle score (HLS) [34]) factors. Dietary factors, including the alternate Mediterranean diet score (aMed) as a dietary quality score [33], dietary intake of calcium, magnesium, sodium, and vitamin D, as well as calcium and multivitamin supplements, and medical information (e.g., estrogen use, diseases or medications related to bone health, years of menopause (for women)), were also collected in the interviews.

### 2.4. Statistical Analyses

Data were checked for normality before statistical analysis. Continuous variables are expressed as the mean value ± standard deviation (SD), and categorical variables are presented as frequencies and percentages. Differences in mean/rank or proportion between the groups of cases and controls were examined with paired *t*-test or paired Wilcoxon rank sum test (when variables were non-normally distributed) for continuous variables and paired chi-square tests for categorical variables.

The estimated values of NEAP and PRAL were adjusted for total energy intake using the residual method [35]. Based on our previous research [31], participants were separately divided into sex-specific tertiles (T1–T3) based on the distribution of energy-adjusted PRAL and NEAP in the controls, with the sex-specific cutoffs applied to the cases. The lowest tertile group was treated as the reference group. Multivariable conditional logistic regression models were used to assess associations between DAL and hip fracture risk, and odds ratios (ORs) with their 95% confidence intervals (CIs) were estimated. We applied a stepwise method for regression models in light of the possible multicollinearity. In addition, we conducted adjustments of potential confounders based on the biological backdrop, results of univariate analysis, as well as a literature review. Mode 1 was the univariate model. In Model 2, adjustments were made for sociodemographic variables: age (years), sex (matched), BMI (kg/m^2^), occupation (1–4 physical workload levels), education level (1–3 levels), marital status (married/cohabitation vs. others), and family income (1–4 levels). In Model 3, additional adjustments were made for lifestyle factors (including smoking (yes/no), passive smoking (yes/no), alcohol (yes/no) and tea drinking (yes/no), physical activity (metabolic equivalent/week), and HLS (0–4 points)), dietary pattern (aMed score, 0–8 points), dietary intake (calcium (mg/d), magnesium (mg/d), sodium (five levels), vitamin D (IU/d)), dietary supplements of calcium (yes/no) and multivitamins (yes/no), and health information (estrogen use (yes/no) and years since menopause (years) for women). Linear trend testing was performed with the median values of each tertile category of DAL as continuous variables used in the logistic regression model. Restricted cubic spline analyses were further performed with four default knots to evaluate the possible dose–response shape of the DAL–fracture association using the conditional logistic regression model with the same adjustments in Model 3.

Subgroup analyses were conducted to test the effect modification or the consistency of the results across age groups (≤65 and >65 years). For BMI, we chose 23 kg/m^2^ as a cutoff value because of its recommendation as a potential public health action point by the WHO [32]. Multiplicative interactions were implemented by a likelihood ratio test before stratification analyses.

A two-tailed *p* < 0.05 was considered statistically significant in all analyses. All statistical analyses were based on the statistical software package SPSS, v25.0 (SPSS Inc., Chicago, IL, USA), and Stata/MP (16.0) software (StataCorp LLC, College Station, TX, USA).

## 3. Results

### 3.1. Study Population Characteristics

The detailed process of participant selection is presented in Supplementary Table S1. A total of 1070 (women, 795; men, 275) pairs of cases and controls were included in the analyses. Participants’ characteristics and selected risk factors are presented in Table 1. The mean (SD) age was 70.8 (7.3) years for cases and 70.5 (7.0) years for controls. The median (IQR) energy-adjusted intake of PRAL and NEAP, respectively, was 23.0 (15.2, 32.4) mEq/d and −7.96 (−8.31, −7.48) mEq/d for cases and 20.26 (7.84, 37.98) mEq/d and −8.13 (−8.59, −7.37) mEq/d for controls. There was a strong positive correlation between NEAP and PRAL. The Pearson correlation coefficients were 0.822 and 0.732 in the controls and cases, respectively (all *p* < 0.001). Compared with the controls, cases tended to have lower levels of education, BMI, physical activity, family incomes, HLS, aMed, and dietary calcium intake, less habitual tea drinking, less use of calcium or multivitamin supplements, and estrogen (in women), and higher proportions of smokers or passive smokers as well as higher DAL values. (Table 1). In the controls, higher NEAP was associated with higher intakes in food groups of grain and meats, but lower intakes of fruit, vegetables, eggs, and dairy foods; there were no significant differences in the intakes of soy foods, poultry, and fish/shellfish (Table 2).

### 3.2. Dietary Acid Load and Hip Fracture Risk

**Figure 1:**
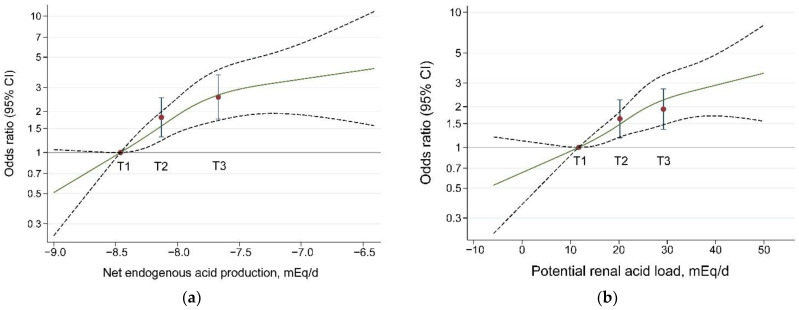
Dose–response associations of the levels of dietary acid load with the risk of hip fracture using restricted cubic spline analyses. (**a**) Net endogenous acid production and hip fracture. (**b**) Potential renal acid load and hip fracture. Restricted cubic splines (RCS) with four knots were used to estimate the associations in conditional regression models. Models were adjusted for potential confounders (see Model 3 in Table 2). The reference value was median level of tertile 1. The solid lines and the red circles refer to estimates of odds ratios (OR); the dashed lines and the error bars represent 95% CI. T1, T2, and T3: tertiles 1 to 3.

Significantly positive associations were observed between dietary acid load (both NEAP and PRAL) and the risk of hip fracture in total participants in all three models (all *p*-trend < 0.001). Compared with the lowest tertiles in Model 3, the ORs (95% CIs) of hip fracture for the highest tertiles were 2.55 (1.76, 3.71), 2.11 (1.42, 3.15), and 1.77 (0.66, 4.71) for NEAP and 1.92 (1.36, 2.71), 1.97 (1.32, 2.92) and 1.31 (0.58, 2.95) for PRAL in total participants, women and men, respectively (Table 3). The dose-dependent associations between DAL (PRAL and NEAP) and the risk of hip fracture were further confirmed by restricted cubic spline analyses after fully adjustment (Model 3) by using the medians of PRAL and NEAP as the referent in total participants (Figure 1). The slope of odds increases related to DAL tended to taper off at DAL levels over the medians of the highest tertile. NEAP showed a more significant association with hip fracture risk than did PRAL. In Model 3, the other major beneficial factors of hip fracture were higher value/level of BMI, income, education, aMed, tea drinking, and use of vitamins and estrogen. In contrast, risk factors included a longer duration of menopause and smoking (data not shown). Model 1 exhibited the most significant results, followed by Models 2 and 3 (Table 3).

### 3.3. Subgroup Analyses

The subgroup analyses showed that the dose-dependent positive associations between DAL and hip fracture tended to be more pronounced in those fracture-vulnerable populations with older age (>65 years), particularly with lower BMI (<23 kg/m^2^) (*p*_-interaction_ < 0.001) (Table 4).

## 4. Discussion

To our knowledge, this current study of 1070 pairs of age- and sex-matched cases and controls is the first to focus on identifying the potential influence of DAL on hip fracture risk in a Chinese population. Our findings revealed that DAL estimated by both PRAL and NEAP had significant positive associations with the risk of hip fracture among middle-aged and older Chinese people. The results supported the hypothesis that a high dietary acid load might have an adverse effect on bone health.

Previous studies suggested a detrimental association of high DAL with bone health [17,18,19,20,21,22]. In a cross-sectional study with 1056 women, NEAP was inversely associated with BMD at the spine and hip [18]. Another cross-sectional study showed a significant inverse correlation between higher NEAP and bone formation in healthy community-based Iranian subjects [19]. Similar associations were also noted in cohort/longitudinal studies. An 8.37-year prospective study including 36,217 French postmenopausal women showed that higher renal net acid excretion was associated with a significantly increased risk of fracture in participants in the lowest quartile of calcium intake [17]. Another 3-year prospective study found that plasma HCO_3_ was positively associated with BMD at both 1- and 3-year follow-ups in 3075 older men and women in the US [20]. A recent meta-analysis showed significantly detrimental associations between NEAP and BMD and between PRAL and fracture risk [36]. An experimental study demonstrated that decreasing NEAP by increasing vegetable and fruit intake might protect against bone loss during spaceflight [37]. A meta-analysis of RCTs suggested that alkaline supplements had beneficial effects on bone density or bone biomarkers [38]. However, null associations between DAL acidic diet and bone health were also noted in some studies, as summarized in the meta-analyses [36,38]. In accordance with the majority of these previous findings, our study also suggested that high DAL was positively associated with an augmented risk of hip fractures. Considering no detrimental effect within usual protein intakes of 0.8–1.3 g/Kg/day [34] and the beneficial role of alkaline supplements in bone health [35], the harmful associations of DAL with fracture suggested the importance of adequate alkaline foods. In this study, the higher dietary acid load was mainly contributed by higher intakes of grain and meats but lower intakes of fruit, vegetables, eggs, and dairy foods (Table 2). Therefore, a dietary pattern with moderate grain and meats but more fruit and vegetables and dairy foods might benefit bone health [32,39].

Several underlying biological mechanisms might be responsible for the positive associations between DAL and hip fractures. On the one hand, a long-term nutritional acid load can result in chronic metabolic acidosis [40,41], which then stimulates physiochemical mineral dissolution and subsequent cell-mediated bone resorption. On the other hand, acidosis inhibits the activity of osteoblasts and reduces specific matrix protein gene expression and alkaline phosphatase activity [42]. Osteoblasts can induce the production of prostaglandins and increase the synthesis of nuclear factor kappa B ligand (NFkb) and its receptor activator (RANKL) in a paracrine manner [43]. These further promote the activity of osteoclasts and the formation of new osteoclasts to promote bone absorption and proton load buffer [44,45].

The literature reported an inconsistent result related to the association between DAL and BMD/fractures [36,38]. The reasons for the between-study/population heterogeneity remained uncertain due to limited studies published. Subgroup analyses in the previous meta-analysis showed that the estimated effect size might vary with study locations, age groups, and varied adjustments [36]. In this study, the subgroup analyses showed that the dose-dependent positive associations between DAL and fracture were significant only in those with lower BMI (≤23 vs. >23 kg/m^2^, *p*-interaction < 0.001). Some studies also found that BMI might modify the associations of some risk factors and bone indices. One study showed that a significant beneficial association between physical activity and calcaneal broadband ultrasound attenuation was evident only in women with BMI < 30 but not in those with BMI ≥ 30 kg/m^2^ [46]. In another study with 3985 postmenopausal Canadian women, frailty-related fracture risks were significantly higher in nonobese women than in obese women (hazard ratio, 1.34 vs. 0.72) [47]. A higher BMI was associated with a lower risk of fractures in both men and women, as reported in a meta-analysis that included 37 cohorts and 38,200 incident fracture cases [48]. Thus, higher BMI might mask or attenuate the associations of other determinants of bone health. In addition, we found that the unbeneficial association between PRAL/NEAP and fracture risk was more evident in people aged beyond 65 years than those ≤65 years. Our finding suggested that the body’s ability to process nutrients might decline with age. In addition, previous evidence revealed that aging is associated with a decline in a number of physiological functions that can affect nutritional status [49,50]. The BMI- and age-specific association of DAL with fracture risk in this study might explain (at least in part) the null association observed in the studies by Jia et al. [26] (higher BMI, around 27 kg/m^2^) and by Dargent-Molina et al. [17] (younger age) as compared with our population. Therefore, it is of more importance to have a balanced diet in people with lower BMI or older generations than those with higher BMI or younger ages.

Our study has a few limitations that warrant mention. First, the case–control study design could not infer a causal relationship due to unclear time sequence between the exposure and outcome and residual confounding. In this study, we tried to avoid reverse causality by excluding patients with substantial changes in dietary habits in the preceding five years and including fracture patients diagnosed within the previous two weeks. To address potential confounding factors, we adjusted for a large variety of important determinants of bone health, including sociodemographic data, body mass index, lifestyle factors, dietary factors, and some medical information. The covariates were selected according to biological relevance and the results of univariate analysis (Table 1). Residual confounding, however, could not be entirely excluded because of some unmeasured factors (e.g., medications) and measurement errors. Second, both PRAL and NEAP are indirect indicators and would be less accurate and precise than the direct measurement of urinary net acid excretion in 24 h urine for the evaluation of dietary acid load [51]. However, the PRAL and NEAP are well-established assessments for DAL with good validity [51,52]. Third, using the FFQ for the estimation of dietary DAL unavoidably led to measurement errors and recall bias, although the FFQ used in our study was verified [53]. Future studies further testing urinary acid excretion or circulation pH level may help confirm current findings. Finally, our study included approximately 17% hospital-based controls, which might be prone to selection bias. Fourth, we did not have the data on BMD and the history of previous fractures in the case group and could not holistically evaluate the effect of dietary DAL on osteoporosis in this study. Considering the less significant associations observed in the subgroup of hospital-based controls than those using the community-based controls, it is likely that the overall association might be underestimated.

## 5. Conclusions

Our findings suggest that higher DAL was positively associated with the risk of hip fractures, especially in those with a lower BMI of less than 23 kg/m^2^. Future prospective studies with objective DAL measures are warranted to confirm our findings.

## Figures and Tables

**Table 1:** Demographic and socio-behavioral characteristics and selected hip fracture risk factors of the study population (1070 pairs).

Variable	Women (795 Pairs)		*p*-Value	Men (275 Pairs)		*p*-Value
	Case	Control		Case	Control	
Age, years	71.1 ± 7.2	70.7 ± 6.9	0.233	69.7 ± 7.6	69.7 ± 7.2	0.982
Body mass index, kg/m^2^	21.8 ± 3.4	23.3 ± 3.0	<0.001	21.5 ± 3.0	23.4 ± 2.6	<0.001
Marital status, *n* (%)			<0.001			<0.001
Married or cohabitation	454 (57.3)	536 (67.6)		230 (83.0)	256 (92.4)	
Others	339 (42.8)	257 (32.4)		47 (17.0)	21 (7.6)	
Education, *n* (%)			<0.001			<0.001
Primary school or below	457 (57.6)	238 (30.0)		101 (36.5)	47 (17.0)	
Junior high school	119 (15.0)	171 (21.56)		57 (20.6)	53 (19.1)	
High school or above	217 (27.4)	384 (48.4)		119 (43.0)	177 (63.9)	
Family monthly income (Yuan/person), *n* (%)			<0.001			<0.001
≤500	62 (7.8)	14 (1.8)		12 (4.3)	1 (0.4)	
501–2000	212 (26.7)	108 (13.6)		48 (17.3)	17 (6.1)	
2001–3000	172 (21.7)	214 (27.0)		50 (18.1)	59 (21.3)	
>3000	347 (43.8)	457 (57.6)		167 (60.3)	200 (72.2)	
Occupation, *n* (%)			<0.001			<0.001
All mental work	196 (24.7)	253 (31.9)		92 (33.2)	88 (31.8)	
Mainly mental work	82 (10.3)	173 (21.8)		35 (12.6)	59 (21.3)	
Mainly physical labor	184 (23.2)	191 (24.1)		70 (25.3)	86 (31.)	
All physical labor	316 (39.9)	158 (19.9)		72 (26.0)	39 (14.1)	
Other	15 (1.9)	18 (2.3)		8 (2.89)	5 (1.8)	
Saltiness of habitual diets, *n* (%)			0.020			<0.001
Light or very light	339 (43.0)	394 (50.0)		73 (26.5)	121 (44.0)	
Moderate	302 (38.3)	271 (34.4)		131 (47.6)	102 (37.1)	
Salty or very salty	146 (18.5)	123 (15.6)		73 (26.5)	52 (18.9)	
Health lifestyle score, *n* (%)			<0.001			0.025
0–1	148 (19.3)	78 (10.0)		49 (18.5)	32 (11.9)	
2	288 (37.5)	220 (28.2)		87 (32.8)	82 (30.4)	
3	263 (34.2)	295 (37.8)		102 (38.5)	109 (40.4)	
4	69 (9.0)	187 (24.0)		27 (10.2)	47 (17.4)	
Alternate Mediterranean diet score, *n* (%)			<0.001			<0.001
0–2	244 (30.8)	114 (14.4)		81 (29.3)	39 (14.2)	
3	182 (23.0)	118 (14.9)		56 (20.3)	49 (17.8)	
4	175 (22.1)	202 (25.5)		59 (21.4)	65 (23.6)	
5–8	191 (24.1)	357 (45.1)		80 (29.0)	122 (44.4)	
Physical activity, MET⋅h/day ^d^	66.7 ± 38.6	82.1 ± 53.1	<0.001	70.6 ± 44.6	77.6 ± 49.1	0.081
Estrogen user, *n* (%)	11 (1.4)	59 (7.5)	<0.001			
Years since menopause, years	21.9 ± 8.3	20.7 ± 8.1	0.002			
Smoker, *n* (%) ^a^	37 (4.7)	15 (1.9)	0.002	143 (51.6)	120 (43.3)	0.060
Passive smoker, *n* (%) ^b^	172 (21.7)	142 (17.9)	<0.001	54 (19.5)	30 (10.8)	0.003
Alcohol drinker, *n* (%) ^c^	20 (2.5)	37 (4.7)	0.022	53 (19.1)	40 (14.4)	0.150
Tea drinker, *n* (%)	252 (31.8)	363 (45.8)	<0.001	119 (43.0)	177 (63.9)	<0.001
Calcium supplement user, *n* (%)	268 (33.8)	367 (46.3)	<0.001	45 (16.3)	88 (31.8)	<0.001
Multivitamin supplement user, *n* (%)	80 (10.1)	227 (28.6)	<0.001	22 (7.9)	80 (28.9)	<0.001
Dietary calcium intake (mg/d)	441 (344, 555)	493 (401, 607)	<0.001	534 (416, 659)	572 (476, 689)	0.004
Dietary magnesium intake (mg/d)	315(271,361)	324(281,372)	0.005	395 (351,440)	407(363,458)	0.013
Dietary vitamin D intake (IU/d)	66.9 (48.5, 92.3)	68.6 (49.8, 96.2)	0.265	82.2 (59.5, 115.3)	85.0 (57.7, 116.9)	0.678
Net endogenous acid production, NEAP (mEq/d)	−8.02 (−8.25, −7.78)	−8.18 (−8.41, −7.90)	<0.001	−7.82 (−8.03, −7.56)	−7.98 (−8.24, −7.70)	<0.001
Potential renal acid load, PRAL (mEq/d)	21.2 (16.7, 24.9)	18.3 (12.9, 23.2)	<0.001	30.2 (27.1, 34.8)	28.0 (22.3, 32.3)	0.006

^a^ Smokers were defined as those who have smoked at least one cigarette daily for at least six consecutive months. ^b^ Passive smoker: defined as exposure to other people’s tobacco smoke for at least 5 min daily during the previous year. ^c^ Alcohol drinker: defined as consuming an alcoholic drink at least once per week for at least six consecutive months at any time during the person’s life. ^d^ Physical activities included daily occupational, leisure-time, and household chores, evaluated by metabolic equivalent (MET) hours per day.

**Table 2:** Mean (SD) intakes of food groups by quartiles of NEAP in the controls (*n* = 1070).

Food Groups	Tertile 1	Tertile 2	Tertile 3	*p*-Trend
	Median (IQR)	Median (IQR)	Median (IQR)	
Grain *	167 (141, 203)	185 (157, 221)	192 (161, 230)	<0.001
Soy foods *	5.4 (2.4, 10.4)	5.2 (2.6, 11.7)	7.9 (2.8, 19.9)	0.090
Vegetables	452 (357, 559)	317.5 (249, 373)	187 (121, 263)	<0.001
Fruits	155.3 (94.8, 204.7)	94.2 (60.4, 133.2)	36.6 (20.2, 70.0)	<0.001
Meats	58.1 (43.5, 79.5)	67.3 (49.6, 93.0)	84.1 (60.6, 126.9)	<0.001
Poultry	16.4 (9.5, 28.3)	19.6 (11.3, 29.0)	17.1 (10.7, 29.5)	0.614
Fish and shellfish	34.9 (20.2, 54.2)	35.2 (22.0, 55.7)	27.0 (12.8, 49.4)	0.196
Eggs	28.4 (19.3, 44.4)	27.2 (18.6, 44.6)	18.5 (11.4, 28.5)	<0.001
Dairy foods *	20.5 (9.2, 31.1)	14.4 (5.7, 22.0)	3.9 (0.6, 9.5)	<0.001

Food intake: energy-adjusted food intake (residuals + mean intake) by sex. *: Calculated in dry form (no water), the other foods calculated in raw material form (edible portion), g/d. IQR: interquartile range.

**Table 3:** Odds ratio (95% CIs) of hip fracture for tertiles of dietary acid load ^a^.

NEAP/PRAL by Sex	Tertiles of Dietary Acid Load			*p*-Trend
	Tertile 1	Tertile 2	Tertile 3 (Highest)	
**Women and men**				
NEAP, mEq/d				
*n* (case/control)	176/356	371/357	523/357	
Median (mEq/d)	−8.46	−8.13	−7.67	
OR 1 ^c^	1.00	2.14 (1.68, 2.72)	3.37 (2.62, 4.34)	<0.001
OR 2 ^c^	1.00	1.82 (1.38, 2.40)	2.73 (2.04, 3.65)	<0.001
OR 3 ^c^	1.00	1.81 (1.30, 2.53)	2.55 (1.76, 3.71)	<0.001
PRAL, mEq/d				
*n* (case/control)	185/356	371/357	514/357	
Median (mEq/d) ^b^	11.7	20.2	29.2	
OR 1 ^c^		2.00 (1.59, 2.53)	3.02 (2.37,3.85)	<0.001
OR 2 ^c^	1.00	1.73 (1.32, 2.27)	2.30 (1.74, 3.04)	<0.001
OR 3 ^c^	1.00	1.63 (1.18, 2.25)	1.92 (1.36, 2.71)	<0.001
**Women**				
NEAP, mEq/d				
*n* (case/control)	140/265	266/265	387/265	
Median (mEq/d)	−8.48	−8.17	−7.75	
OR 1 ^c^	1.00	1.93 (1.47, 2.54)	3.07 (2.31, 4.08)	<0.001
OR 2 ^c^	1.00	1.56 (1.14,2.15)	2.27 (1.63,3.17)	<0.001
OR 3 ^c^	1.00	1.60 (1.11,2.32)	2.11 (1.42,3.15)	0.002
PRAL, mEq/d				
*n* (case/control)	140/265	264/265	389/265	
Median (mEq/d) ^b^	10.5	18.4	25.1	
OR 1 ^c^	1.00	1.91 (1.45, 2.50)	3.07 (2.31, 4.08)	<0.001
OR 2 ^c^	1.00	1.84 (1.33,2.54)	2.34 (1.67,3.27)	0.001
OR 3 ^c^	1.00	1.76 (1.21,2.56)	1.97 (1.32,2.92)	0.005
**Men**				
NEAP, mEq/d				
*n* (case/control)	36/91	105/92	136/92	
Median (mEq/d)	−8.31	−7.97	−7.57	
OR 1 ^c^	1.00	3.31 (1.93, 5.69)	4.81 (2.74, 8.43)	<0.001
OR 2 ^c^	1.00	2.22 (1.15,4.29)	2.70 (1.39,5.23)	0.034
OR 3 ^c^	1.00	1.86 (0.76,4.54)	1.77 (0.66,4.71)	0.164
PRAL, mEq/d	20.1	28.3	34.9	
*n* (case/control)	45/91	107/92	125/92	
Median (mEq/d) ^b^	11.7	20.2	29.2	
OR 1 ^c^	1.00	2.39 (1.51, 3.79)	2.94 (1.82, 4.75)	<0.001
OR 2 ^c^	1.00	1.36 (0.74,2.50)	2.03 (1.12,3.68)	0.035
OR 3 ^c^	1.00	1.12 (0.50,2.50)	1.31 (0.58,2.95)	0.459

^a^ Tertiles based on the tertile cutoffs of dietary acid load in the controls by gender. ^b^ Median intake of dietary acid load in the controls. ^c^ OR: odds ratio and 95% CIs from the conditional logistic model by the stepwise method. OR 1: crude OR; OR 2: age, gender, BMI (kg/m^2^), occupation, education level, marital status, and family income were adjusted for; OR 3: we further adjusted for lifestyle factors (such as smoking, passive smoking, alcohol and tea drinking, physical activity, aMed score, HLS) and dietary intake (calcium, magnesium, sodium, vitamin D), dietary supplements of calcium and multivitamins, and health information (estrogen use, years since menopause).

**Table 4:** Odds ratio (95% CIs) of hip fractures for tertiles of dietary acid load by subgroups.

Subgroups	PRAL			*p*_-Trend_	*p*_-Interaction_	NEAP			*p*_-Trend_	*p*_-Interaction_
	T1	T2	T3 (Highest)			T1	T2	T3 (Highest)		
Age, year					0.091					0.368
≤65	1.00	1.02 (0.58, 1.78)	0.94 (0.53, 1.66)	0.807		1.00	1.62 (0.91, 2.88)	1.38 (0.75, 2.54)	0.036	
>65	1.00	1.84 (1.33, 2.56)	2.32 (1.66, 3.25)	<0.001		1.00	1.71 (1.23, 2.34)	2.54 (1.78, 3.63)	<0.001	
BMI, kg/m^2^					0.005					<0.001
<23.0	1.00	1.65 (1.13, 2.56)	2.33 (1.58, 3.46)	<0.001		1.00	2.06 (1.40, 3.12)	3.48 (2.30, 5.27)	<0.001	
≥23.0	1.00	1.91 (1.25, 2.91)	1.47 (0.93, 2.31)	0.152		1.00	1.95 (1.27, 2.98)	1.57 (0.96, 2.56)	0.221	

Odds ratios (95% CI) were from multivariate conditional logistic regression models. Covariates adjusted as Model 3 in Table 2.

## Data Availability

Not applicable.

## Supplementary Materials

The following supporting information can be downloaded at: https://www.mdpi.com/article/10.3390/nu14183748/s1, Supplementary Table S1: Process of participant selection.
