# Supplementary material for: Dietary Acid Load Was Positively Associated with the Risk of Hip Fracture in Elderly Adults
Source: Nutrients. 2022 Sep 10;14(18):3748. doi: 10.3390/nu14183748 (PMC9503794; doi:10.3390/nu14183748)
Supplement: Supplementary file 1 [file nutrients-14-03748-s001.zip › nutrients-1905506-supplementary.pdf]

Supplementary Table S1. Process of participant selection.

Procedures of Participant Selection	Cases	Matched Controls	
		Community-Based	Hospital-Based
Screened	1915	1608	307
Excluded (in total)	845	721	124
Diseases affecting dietary habits	352	351	52
Diseases affecting routine activities	191	242	22
Pathological or high-energy fractures	96	0	0
Unable to communicate	43	28	10
Refused to participate	138	5	20
Unreasonable energy intakes*	25	21	7
History of any fracture	0	74	13
Included in the analyses	1070	887	183

*Reasonable range: 800–4000 kcal/d for males and 500–3500 kcal/d for females.
